# A Study of Innovative Features in Scholarly Open Access Journals

**DOI:** 10.2196/jmir.1802

**Published:** 2011-12-16

**Authors:** Bo-Christer Björk

**Affiliations:** ^1^Hanken School of EconomicsHelsinkiFinland

**Keywords:** Scholarly publishing, open access, Internet, peer review

## Abstract

**Background:**

The emergence of the Internet has triggered tremendous changes in the publication of scientific peer-reviewed journals. Today, journals are usually available in parallel electronic versions, but the way the peer-review process works, the look of articles and journals, and the rigid and slow publication schedules have remained largely unchanged, at least for the vast majority of subscription-based journals. Those publishing firms and scholarly publishers who have chosen the more radical option of open access (OA), in which the content of journals is freely accessible to anybody with Internet connectivity, have had a much bigger degree of freedom to experiment with innovations.

**Objective:**

The objective was to study how open access journals have experimented with innovations concerning ways of organizing the peer review, the format of journals and articles, new interactive and media formats, and novel publishing revenue models.

**Methods:**

The features of 24 open access journals were studied. The journals were chosen in a nonrandom manner from the approximately 7000 existing OA journals based on available information about interesting journals and include both representative cases and highly innovative outlier cases.

**Results:**

Most early OA journals in the 1990s were founded by individual scholars and used a business model based on voluntary work close in spirit to open-source development of software. In the next wave, many long-established journals, in particular society journals and journals from regions such as Latin America, made their articles OA when they started publishing parallel electronic versions. From about 2002 on, newly founded professional OA publishing firms using article-processing charges to fund their operations have emerged. Over the years, there have been several experiments with new forms of peer review, media enhancements, and the inclusion of structured data sets with articles. In recent years, the growth of OA publishing has also been facilitated by the availability of open-source software for journal publishing.

**Conclusions:**

The case studies illustrate how a new technology and a business model enabled by new technology can be harnessed to find new innovative ways for the organization and content of scholarly publishing. Several recent launches of OA journals by major subscription publishers demonstrate that OA is rapidly gaining acceptance as a sustainable alternative to subscription-based scholarly publishing.

## Introduction

### Development of Scientific Journal Publishing

The scientific journal as an institution dates back to the late 17^th^ century. Until the Second World War, scholarly journals were mainly published by scientific societies, and subscriptions were primarily individual and often linked to society membership. After 1950, the number of journals increased rapidly, and commercial publishers entered the market to meet the increased demand for outlets. Today there are almost 30,000 peer-reviewed scholarly journals indexed in Ulrich’s periodicals directory, and there are several thousand journals more, particularly journals published in languages other than English. Approximately 1.5 million articles are published yearly [1].

From the perspective of the scientific community as a whole, the scholarly journal fulfills a number of functions [2]. Gierveld, for instance, names four such functions: current awareness, archival recording, priority claim, and quality control [3]. Over the years, the format of scientific articles hasn’t changed much. In most disciplines, articles are a few pages long and are bundled into regularly appearing issues that are collected into yearly volumes. Citations provide the “glue” that links the articles into the context of a scientific field’s body of knowledge. The quality assurance mechanisms have undergone a gradual change as the anonymous peer-review process evolved into an “industry standard” in the 20^th^ century [4].

During the past two decades, the scientific journal publishing process has undergone more change than during the preceding three centuries together. Today, almost all major subscription journals are available in both paper and electronic formats, giving academics at major universities instant access to thousands of journals, mainly via bundled e-licensing agreements with the major publishers. Publishers have also adopted Web-based manuscript and review management systems. From the readers’ viewpoint, a very significant improvement is the emergence of general Web search engines, as well as specialized services dedicated to scientific literature (ie, Google Scholar), which facilitate discovering and tracking publications enormously.

The scientific publishing industry has a peculiar oligopolistic structure, which has created extremely high barriers for new entrants and has ensured the major publishers a high level of profitability [5]. Because of this situation, mainstream publishers have had little incentive to experiment with the radical innovation of open access (OA), and part of the potential of the Web for dissemination of scientific knowledge has remained untapped. Instead, individual scholars and new start-up publishers have taken the initiative and have in a short time launched several thousand OA journals.

### Open Access

The fundamental principle of open access, that the results of science should be openly accessible to anybody, is perfectly in harmony with the fundamental ethos of science and also with the interests of authors, academic institutions, and research funders. Open access can be achieved in two ways: via direct electronic OA publishing (ie, gold OA) or, alternatively, by publication in traditional subscription journals combined with parallel posting of the manuscript openly on the Web (ie, green OA) [6]. This paper deals only with direct OA. There are several comprehensive studies of both routes to OA [7-9].

A recent study has estimated that the number of OA journals increased by 500% and the number of articles by 900% during the decade 2000-2009 [10]. The difference between the two growth measures is explained by the fact that the average yearly number of articles published per OA journal rose from around 20 to 40 during the period. In 2009, there were around 4800 active OA journals, which published approximately 190,000 articles. An estimated 7.7% of all peer-reviewed articles were published in full OA journals [10].

Behind these aggregate numbers, the population of OA journals is very heterogeneous in size, funding mechanism, Web features, and the method of peer review and scientific quality. The academics and publishers behind these journals have experimented with many of the parameters of scholarly journal publishing, sometimes successfully, sometimes ending in failure. So far, there have mainly been reports about individual OA journals [11-15] focusing on the features and experiences gained from the journal in question. In this study, we attempt to paint a picture of the broader spectrum of these innovations and to draw some tentative conclusions as to where scholarly OA publishing is moving.

### Innovation and Scholarly Publishing

Innovations typically occur in transition periods when technical inventions such as the printing press, steam power, electricity, or the Internet radically change the production conditions and cost structures of whole industries, enabling entrepreneurs to start offering new products or services.

There is a rich literature on the concept of innovation. Tidd et al [16] discuss the “4Ps” of innovation, from a company’s business model perspective. The 4Ps are: (1) product innovation, that is, changes in the products/services which an organization offers; (2) process innovation, that is, changes in the ways in which these products/services are created and delivered; (3) position innovation, that is, changes in the context in which the products/services are introduced; and (4) paradigm innovation, that is, changes in the underlying mental models that frame what the organization does.

Of these categories, product, process, and paradigm innovation are easily applicable to our context. Although a peer-reviewed journal article in its traditional printed format can be seen as a product, it is more useful to view the publication of scholarly journal articles as service provision, since the product is not consumed when read and the key issue is disseminating the information as efficiently as possible to potential readers. The process perspective is also important since both electronic publishing and open access publishing enable major changes in the process [17,18]. The paradigm innovation in the context of publishing are the ideas of making the journals available to the whole world for free and of funding the service by means other than charging the readers.

Baregheh et al [19] define innovation as “the multistage process whereby organizations transform ideas into improved products, services, or processes in order to advance, compete, and differentiate themselves successfully in their marketplace.” This definition stresses that the driving force of innovation is to improve the competitive position of market players, which leads to the concept of a business model. For our purposes, a useful definition is: “The essence of a business model is that it defines the manner by which the business enterprise delivers value to customers, entices customers to pay for value, and converts those payments to profit: it thus reflects management’s hypothesis about what customers want, how they want it, and how an enterprise can organize to best meet those needs, get paid for doing so, and make a profit” [20]. Since many of the publishers of scholarly journals are scientific societies, groups of independent scholars, and so on, this definition should be extended from a commercial profit motive alone to also ensuring the long-term economic sustainability of the publishing operations.

A key element in this definition is the concept of *value to customers*. A scientific journal can only be successful and sustainable if it succeeds in delivering value appreciated by its customers and in covering the costs of its operations by monetary income or voluntary efforts. In the open access context, the authors are the key customers. One could argue that the editors and reviewers should be included as customers as well, or perhaps more appropriately, as partners. Editors and reviewers make very significant contributions, in particular, they contribute to journals with rigorous peer review in exchange for the personal network and the prestige they gain within their academic communities by being associated with the journal and are hence receiving a sort of service from the journal in exchange for their value-added work. The success of any start-up peer-reviewed journal is very much dependent on attracting this type of contributor.

The effects of the Web on scholarly publishing can be seen as consisting partly of effects enabled by the e-infrastructure as such and those enabled by opening up the e-versions with no access restrictions. In an industry with high barriers to the entry of new companies, established journal publishers, in the first instance, have striven to use the medium of the Internet to enhance the current business model. The more radical innovation of opening up the Web versions of journals has forced new journals and publishers to come up with alternative business models at the same time as it has offered the chance of offering a different type of service to authors. This innovation can be compared with radical changes in other information-related industries, as exemplified by successful companies and community services like Skype, Wikipedia, and Red Hat.

A useful two-dimensional framework for discussing the development in scholarly publishing during the last 15 years can be constructed using the principles of dissemination technology and access (Table 1).

**Table 1:** A typology of scientific peer-reviewed journals

	Restricted Access	Open Access
Paper only	Traditional printed journal	-
Paper and electronic	All major publishers today	Immediate or delayed OA to electronic version
Electronic only	Very rare type of journal	Full OA journal

The position of a journal in this framework defines many of the border conditions for the features a journal can experiment with. Until the emergence of the Internet, paper printing was the only option, and, in that mode, restricted access for buyers and subscribers was the only viable alternative. (For a brief period, CD-ROM, which can only function in the restricted access mode was also tried, but this was more common for conference proceedings).

*Paper and electronic* is the dominant solution today, as almost all major publishers have launched parallel electronic versions of their journals. Publishers of journals with parallel electronic versions have, in general, restricted access to the e-versions, which has facilitated two new types of distribution mechanisms: the bundled e-licenses, with sometimes over a thousand titles, and the e-commerce, with individual articles on a pay-per-view basis. Since the electronic versions are usually just copies of the articles in the print issues, the structure of journals with a fixed number of regular issues has usually been retained. Many publishers nowadays post accepted and processed papers on the electronic journal sites well in advance of the actual publishing in order to speed up the dissemination, which is otherwise slowed down by articles queuing in line for a fixed number of yearly issues. Other features the electronic medium has made possible are citation linking (ie, Crossref) and alerting emails that contain tables of content and other notifications. Most journals nowadays use electronic manuscript handling systems (either proprietary or open-source), which facilitate the peer-review process without changing the process or the end product, only making the process more efficient.

Some publishers, in particular professional society publishers, have opened up access to the e-versions, which can be accessed for free, directly, or with a delay. This free access is subsidized by income from the print versions or from subscribers wishing immediate access. This is in line with the fundamental purpose of such societies, which is the efficient knowledge dissemination in their subject area. Societies can also see the offering of free e-versions as a way to attract new members and of branding themselves.

The restricted access electronic-only journal is still quite rare. It is well suited for newly founded high volume journals, for instance, which include data sets or case reports.

The last option is the full-fledged open access journal, most of which were “born OA,” which has more freedom than journals in the other categories, with the exception of the revenue model, where readers cannot be charged. So far, electronic-only OA journals have been published mainly by individual academics or start-up OA publishing companies, which tend to use article-processing charges to fund their operations.

The starting point of our further discussion of innovations in scholarly journal publication is the realization that delivering value to the author is what primarily matters for the success of a journal. The collection of article processing charges is only possible if the authors (and, in increasing cases, their funders) perceive that they get value for their money. And this value is in turn dependent on the type of service the journal provides including how widely articles are read and also the branding the journal offers in terms of prestige for the author. Central services that authors are seeking, and which provide them with value, include the prestige of being published in a highly regarded journal, the assurance of being widely read by the relevant readership, speed of publication, and high likelihood of acceptance [21-23].

What kind of new features, then, can open access journals offer that haven’t been possible in traditional journals? Textbox 1 contains a non-exhaustive list of some features.

Some of these features can also be present in subscription-based journals, although they are more commonly found in OA journals.

**Features of Open Access Journals That Differ From Traditional Journals**

Paradigm:

- The universal accessibility per se

Process:

- Cost savings by the use of volunteers for tasks other than peer review
- Cost savings by the use of open-source software
- Cost savings by the use of third party e-portals

Revenue:

- Funding by article processing charges

Product/service:

- Broader or narrower journal topics due to the global reach
- Novel peer review methods
- Faster article publication cycles
- More flexibility in the layout and structure of articles
- Interactivity for after-publication discussions
- Easy reusability of the (digital) content

## Methods

Choosing 20 to 25 journals randomly (or even using a stratified random sample) from the 5000 journals in the DAOJ at the time the study was started would probably not have yielded a very interesting set of journals to study. The vast majority of OA journals consist of individually created journals published by academics, universities, or scientific societies and typically do not use article processing charges for funding [24]. They also tend to use traditional peer-review methods and the articles look much like paper ones. The major innovation is thus the open accessibility itself, not further innovations made possible by the combination of OA and electronic delivery.

A different strategy is to choose key or outlier cases, which have characteristics making them either highly representative or atypical. In theory, the websites of all open access journals listed in the Directory of Open Access Journals (DOAJ) [25] could have been visited and the journals could have been classified according to a predefined list of features. Based on this search, interesting cases could have been identified in a systematic fashion. The list of features would probably have grown from an initial one, as new features would have emerged during the search. For all practical purposes, such a search would have been extremely resource demanding and was ruled out from the start.

Instead, the case journals were identified based on a literature search of articles and conference presentations about open access and also based on the author’s previous extensive knowledge of OA publishing and his personal network. The aim was both to find highly representative cases (where one case is used to represent a large number of journals with fairly similar characteristics) and to find rare atypical cases where journals have experimented with new features. Some journals published in languages other than English were included. The cases also span different revenue models and different sizes ranging from a few articles per year to thousands. The process of case selection was also iterative in the sense that additional interesting candidates came up during conversations with stakeholders or during the study of already selected cases.

In an earlier study, our research group proposed a periodization of the development of OA journals into three periods: a pioneering stage from 1993 through 1999, an innovation period from 2000 through 2004, and a consolidation period beginning in 2005 [10]. This periodization partly influenced the choice of case journals so that each period was represented by several journals. Some basic data about the case journals is shown in Table 2.

For each included journal, information was found using secondary sources (ranging from blog discussions, conference presentation material, and general newspaper items to articles published in peer-reviewed journals), by studying the journal website itself, and by using journal indexes (ie, from the Institute for Scientific Information [ISI] journal citation reports).

There were two options for how the following narrative could have been structured: by journal or by innovative feature. The first option seemed more natural since it would enable the context around the journals to be presented first. In the second option, the case journals would be loosely grouped under a number of dominant themes. The early journals tend to be presented first with the latest newcomers towards the end, but the order is not rigorously followed. The conclusions section is, on the other hand, structured according to the innovations discussed.

**Table 2:** The journals discussed in this paper listed according to the start year of OA publishing.

Journal	Year OA Began	Type of Journal	Type of Publisher	APC (USD)	Impact Factor	Information Technology (IT) Platform	Number of Articles in 2010^a^
*Elore*	1994	Born OA	Society		-	OJS	9
*Journal of Electronic Publishing*	1995	Born OA	University	-	-	Own	20
*Information Research*	1995	Born OA	Scholar	-	0.4	OJS	32
*Medical Education Online*	1996	Born OA	Commercial	800	-	OJS	21
*Electronic Transactions on Artificial Intelligence*	1997	Born OA	Scholar	-	-	Own	ceased
*The International Journal of Design Computing*	1997	Born OA	Scholar	-	-	Own	ceased
*British Medical Journal*	1998	E-version OA	Society	-	13.6	Publisher's	∼1300
*Journal of Medical Internet Research*	1999	Born OA	Scholar	1990	4.7	Own (OJS fork)	64
*Malaria Journal*	2002	Born OA	Commercial	1775	2.9	Publisher's	360
*Journal of Negative Results in Biomedicine.*	2002	Born OA	Commercial	1665	-	Publisher's	9
*PLoS Biology*	2003	Born OA	Non-Commercial	2900	12.9	Publisher's	∼200
*BMC Medicine*	2003	Born OA	Commercial	2265	3.9	Publisher's	60
*Hydrology and Earth System Sciences*	2004	Born OA	Commercial	per page	2.4	Publisher's	∼300
*Advances in Difference Equations*	2004	Born OA	Commercial	600	0.8	Publisher's	133
*Nucleic Acids Research*	2005	Converted to OA	Univ. Press	2770	7.4	Publisher's	∼1200
*African Journal of Food, Agriculture, Nutrition and Development*	2005	E-version OA	Society	-	-	Bioline	111
*PLoS ONE*	2006	Born OA	Non-Commercial	1350	4.3	Publisher's	> 7000
*Diagnostic Pathology*	2006	Born OA	Commercial	1670	1.4	Publisher's	83
*Open Medicine*	2007	Born OA	Scholar	1235	-	OJS	24
*Boletim do Museu Paraense Emílio Goeldi*	2007	E-version OA	Society	-	-	Scielo	27
*Open Information Science Journal*	2008	Born OA	Commercial	800	-	Publisher's	0
*International Journal of General Medicine*	2008	Born OA	Commercial	1865	-	Publisher's	50
*Acta Crystallography: Structure Reports Online*	2008	Converted to OA	Society	150	0.4	Publisher's	> 4000
*Human Genomics and Proteomics*	2009	Born OA	Commercial	1665	-	Publisher's	6

^a^ The numbers of articles for 2010 have been determined by checking the journal websites. In the case of the larger journals, the numbers of articles are approximations.

## Results

### Journals Born as Open Access Founded by Individual Academics

The majority of journals that started publishing as open access during the mid- and late-1990s were new electronic-only journals (often with *electronic* or *online* as part of the name) founded by individual academics or groups of academics. The setting up of a new electronic-only OA journal was simple and required little infrastructure and capital particularly since there was no need for marketing to get subscribers. The central asset was the personal network of the editor, needed to recruit a credible editorial board and to solicit the first submissions. Usually the journals were hosted on the website of the editor-in-chief’s university with home-crafted simple static Web pages. The management of the journal and the peer-review process were usually done on a voluntary basis, and the way the journals were operated was in spirit close to the way many open-source software development projects worked. The volume of manuscripts that were handled was usually rather low.

*Medical Education Online* is a good example of a pioneer volunteer-based OA journal [13]. The journal was from the start (1996) envisaged as a sort of portal for experts interested in medical education and also contained material other than peer-reviewed articles (ie, short discussion items, book reviews, and resource sections where academics could upload material), but over the years, the journal material has been more and more concentrated on articles. Accepted articles are published as they become ready rather than in regular issues, which speeds up publication. The look and feel of the articles is nevertheless exactly the same as in traditional scholarly paper journals.

*Medical Education Online* was originally launched with a number of invited articles, and for the first five years, the number of submissions and published articles was low. But after having survived the first critical years, the numbers have increased (currently around 20 published articles per year), and the journal has established itself within its research community.

For the first decade *Medical Education Online* was published using a Web platform programmed by the editor-in-chief. Over the years, the platform was improved to include, for instance, the possibility for electronic submission of manuscripts. Due to the increase in the workload, the journal adopted article- processing charges (APCs) in 2008 in order to generate a modest revenue. From the start of 2010, the journal has been published by a company specialized in open access publishing (Co-Action Publishing) and uses the Open Journal Systems (OJS) software, a widely used open-source solution for publishing scholarly journals and handling the review process [26]. The level of the APCs has been gradually raised to the current US $800 in order to cover the costs of professional copyediting and the costs of using a professional publishing firm.

The *Journal of Electronic Publishing*, founded in 1995, has had a slightly different development path. After some struggling first years, the journal was on hiatus for four years (2002-2005) before it reemerged and is now published by the University of Michigan Library [27]. Due to the sponsorship from the publishing organization, it has been able to avoid requiring article-processing charges.

*Elore*, is the oldest open access journal from Finland and is a good example of how scientific publishing in languages other than English can benefit from OA. It is published by the Finnish Folklore Society and operates with a minimal budget mainly using volunteer labor. Like many other similar journals, it has recently opted to take into use the OJS software. It publishes articles in both the national languages, Finnish and Swedish, but also publishes articles in English and includes items other than peer-reviewed articles.

Due to the strategic importance of maintaining the scientific discourse in national languages and promoting the local culture, governments and ministries in many countries are providing grants to support local scientific journals, particularly in the social sciences and humanities, where subscription journals are also struggling to make ends meet. In Finland, a problem for a long time was that these grants were based on a percentage of a journal’s monetary income (usually from subscriptions), thus effectively excluding many OA journals from being eligible. Since 2006, the rules have been relaxed, and *Elore* has also benefitted from a small government grant.

In Canada, the Social Sciences and Humanities Research Council (SSHRC) has recently changed its rules for supporting scholarly journals so that the subsidy is Can $850 per published article, with a total maximum of Can $30,000 per journal and a ceiling of Can $5000 for paper or e-distribution costs. These regulations focus the support on the peer-review and copyediting costs of the journal production process, which means that OA journals get an equal treatment compared with paper journals. A very successful OA journal that has received a grant from SSHRC is the Toronto-based *Journal of Medical Internet Research* [15]. The *Journal of Medical Internet Research* is also a forerunner in experimenting with different sources of revenues, including submission fees in addition to charges for published articles, institutional memberships covering APCs of employees, fast-track handling of manuscripts for an extra fee, and sales of the PDF full text versions (the hypertext markup language [HTML] versions are OA) [15]. The journal also experiments with novel methods of peer-review (open peer review) and social media-based article level impact metrics (see Editorial in this issue). 

### Experimenting With Formats and Peer Review

In the mid-1990s, publishers of electronic journals assumed that most readers would prefer to read the articles on screen and would also prefer a straightforward HTML format for the articles, which, for instance, allowed direct hyperlinks to external Web references. Later on, many OA journals chose to format the articles as PDF files, which look like traditional articles in the printout format and which can be easily generated from word processing manuscripts. For the first decade, *Medical Education Online* published HTML and PDF versions in parallel, but since download statistics indicated that readers increasingly preferred PDF versions, the HTML format was dropped after 2005. *Information Research* [11], on the other hand, is still published in the HTML format although it has recently adopted OJS for managing the review process and the article archive.

The *International Journal of Design Computing* (published between 1997 and 2003) dealt with a subject matter (architecture) where the possibility of including high class graphics, three-dimensional models, videos, and even virtual reality simulations in the material was expected to offer an important added value for readers. Like many other early OA journals, the *International Journal of Design Computing* dwindled after the first few years due to a lack of submissions. Academics seemed at that time reluctant to submit their best articles to new experimental electronic-only journals.

*Diagnostic Pathology* (begun in 2006) is another example of a journal trying to use the potential of the electronic medium. Authors can include virtual pathology slides with their articles, and readers can navigate in these with an easy-to-use viewing tool.

In the early years, there was also a lot of enthusiasm about trying out novel forms of peer review and commentary, which the Web enabled. The *Electronic Transactions on Artificial Intelligence* [28] experimented with a process in which authors first uploaded their manuscripts to the journal site followed by a period of open commentary by readers. After a few months, the author could request a formal anonymous peer review of the original submission or an improved version of the manuscript. If the article passed this peer review, which also took into consideration reader comments, it would then receive the status of published journal paper, but the results would have been disseminated earlier. The commentary from readers was also stored with the texts. This type of open peer review represents the linking of the functionality of a subject-based repository for preprints (such as arXiv for physics [29]) or the working paper tradition in disciplines like economics with a single peer-reviewed journal. Other early OA journals that have experimented with open peer review include the *Journal of Medical Internet Research* [15], discussed above. 

In 2004, Copernicus Publications established the publication series *Hydrology and Earth Systems Science Discussions* as a complement to the existing journal, *Hydrology and Earth Systems Science* [30]**.** The idea is that discussion papers can be published within a few days in *Hydrology and Earth Systems Science Discussions* after only a very cursory screening by editorial board members. After that, reader comments and formal peer reviews are openly posted together with the discussed manuscripts. Those that pass the formal peer review are eventually published as full papers in *Hydrology and Earth Systems Science*. Currently, a dozen Copernicus journals use the same structure of twin journals and discussion forums. Copernicus is also interesting for its revenue model since it uses page charges for unrefereed manuscripts published in the discussion sections but publishes the ultimately accepted articles for free.

An additional way in which the Internet can be used to increase the transparency of the peer-review process is to upload the full prepublication history of the manuscript (the reviewers comments and the revisions of the manuscripts) together with the published article, a feature of *BMC Medicine*.

Postpublication peer review is currently being tried out by *Open Medicine*, which has started posting articles on wikis, open to changes and additions by readers [31]. The articles are of the review type and have first undergone a standard peer review before being posted. After that, readers can make changes and additions and also monitor changes and the document history. The idea is close to the idea behind Wikipedia articles, with the major difference being that the original seed document is of a peer-reviewed standard. Review articles are particularly suited to this type of treatment, since the state of the art is continuously changing as new research is being published.

Academics seem to be rather conservative in their choice of publication forums, particularly concerning peer-reviewed articles that are central elements in their publication lists. Due to this, the vast majority of open access journals still adhere to a rather conventional format, and peer-review practices remain largely unchanged.

### Society Journals That Have Made the Electronic Version OA

A relatively low-risk route to OA has been for well-established printed subscription journals to make their electronic versions openly available. Very often the decision to do so has been taken at the time when the e-versions were first made available. One of the pioneers was *British Medical Journal* **,** which started making its research articles openly available in 1998. *British Medical Journal* has a lot of advertising revenue, which is not affected by the decision, and it also offers other material, which is only open to subscribers.

The open e-version strategy has appealed in particular to society journals, which often are using electronic platforms from third parties. Strong society publishers have judged that they have a relatively stable subscription base and other income so that their subscription revenue would not suffer significantly, and they have at the same time been convinced of the service OA can offer the research community.

The leading third party e-portal for American and European society journals is Highwire Press. Among the 1527 journals currently using the platform, 282 offer delayed OA (usually by a year), including very high impact journals such as the *Journal of the American Medical Association*, *Brain*, and *European Heart Journal*. Another 48 of these 1527 journals offer immediate OA.

Outside the Anglo-American sphere, different types of e-portals have emerged. These portals are directly or indirectly government-sponsored and have a mission to help local scholarly journals reach a wider global audience. In a sense, they provide a form of subsidy for journals that choose to make their e-versions open access since their use is usually free provided that the journals fulfill scholarly criteria. Due to the economies of scale, these services are in fact quite cheap compared with the journals themselves setting up e-versions. Packer [32], for instance, mentions that the cost per published article is US $60 for the Scientific Electronic Library Online (SciELO) portal.

Such portals are very important in the Spanish and Portuguese speaking countries (SciELO, Red de Revistas Cientificas de América Latina y el Caribe **.** [Redalyc]) [33] and in Japan (Japan Science and Technology Aggregator, Electronic [J-STAGE]) [34]. In total, these three portals alone contribute around 14% of all OA journals listed in DOAJ. A recent study has shown that of all the roughly 15,000 peer-reviewed journals indexed in 2010 in Scopus, the percentages that were OA were 73.9% for Latin America, 4.9% for North America, and 6.9% for Europe [35], clear evidence of how widely established high-quality Latin American journals had made their e-versions openly available via such portals.

As an example of the effects of such portals, consider the journal *Boletim do Museu Paraense Emílio Goeldi: Ciências Humanas*. This journal has its roots in one of the oldest scholarly journals in Brazil (established in 1894) and publishes articles in the social sciences and humanities with topics related to the Amazonas region. The language of the articles in the journal is Portuguese, but all articles also have abstracts in English. In fact, 15 years ago, finding out about articles in the journal would have been very difficult unless the reader belonged to a very select group of people who either had a personal subscription or their university happened to subscribe to and archive the paper journal. Since the electronic full text version of this article is now openly available via Scielo, anybody with Internet access who might take an interest in this sort of topic will now easily find it, for instance, via a Google keyword search or tracking a reference found in another publication.

Another example of the positive effects of third party OA portals on bridging the digital divide is University of Toronto-based Bioline International, which explicitly aims at helping journals in developing countries publish electronic OA versions. Bioline finances its operations via sponsorship and supporting members. One of the 54 journals on Bioline’s website is the *African Journal of Food, Agriculture, Nutrition and Development* published by the Rural Outreach program based in Kenya. The journal also has a print edition, but has been available since 2005 in an electronic OA version via Bioline. Considering the global challenges in feeding the world’s inhabitants, the journals’ articles can be of interest to a wide audience in academia, government, and international organizations.

### The Emergence of Specialized OA Publishers

BioMed Central (BMC), founded in the year 2000 by Vitek Tracz, was the first specialized professional OA publisher. Since 2002, the business model of BMC has been to fund operations mainly with article processing charges (APCs) and to launch a large number of journals in different fields of biology and medicine to benefit from economies of scale in e-infrastructure, marketing, and so on. BMC was eventually successful enough to attract the large mainstream publisher Springer to buy the company in 2008. In 2010, BMC’s 234 journals published more than 15,000 articles.

An interesting example of a BMC journal is the *Malaria Journal* **,** which publishes research on topics of vital interest to researchers and practitioners in the developing world, who often have problems financing subscriptions to the research literature. Hence, open access is of particular importance. BMC, as most OA publishers, waives the APCs (currently US $1775 for *Malaria Journal*) for authors who have problems getting funding for this, in particular authors from developing countries.

Another BMC journal with an innovative scope is the *Journal of Negative Results in Biomedicine* [36]. Over the nine years of its existence, the journal has published few articles and thus cannot be considered successful, but probably due to its low marginal costs and APC revenue, it is still operating.

Public Library of Science was originally mainly a Web campaign promoting open access. When the campaign failed to have the intended impact, the originators together with Harold Varmus, a Nobel Prize winner and former director of the National Institutes of Health, founded an OA publishing company, also named Public Library of Science (PloS). Thanks to a substantial initial grant of US $9 million, the company was able to launch two very high quality journals in 2003-2004 and has since expanded to seven journals. *PLoS Biology* currently has the highest ISI impact factor (12.9) of all general biology journals. In addition to the fast peer-review and publishing schedules typical for OA journals, PLoS has strived to offer both authors and readers articles with high-class layout and interactive features, including download statistics and reader comments.

Both BioMed Central and PloS publish journals mainly in biomedicine, a segment of science where research funding is abundant and where authors (through their institutions) can usually afford to pay the APCs. Other OA publishers try to cover all fields of science with their journal portfolios. The publisher Hindawi is an interesting case, since it operates from Egypt and has been able to keep publishing costs down due to much lower personnel costs [14,37]. Despite this, its operations are fully global. Hindawi was founded as a conventional publisher in 1997 but started to convert journals to OA financed with APCs in 2003, and four years later, all of its journals were OA. A good example of Hindawi’s journals is *Advances in Difference Equations*. For a journal in mathematics, the peer review and copyediting can be quite labor-intensive, but the APC is still quite reasonable at US $600. The journal is a popular outlet for mathematicians from a wide spectrum of countries, and its global reach is well reflected in the composition of its scientific editorial staff and editorial board.

The picture of open access publishers wouldn’t be complete without a discussion of Bentham and Dove Press, both of which have created controversy in the OA publishing debate [38,39]. Bentham massively launched over 200 OA journals in 2007 under the label Bentham Open. In connection with the launch, academics around the world were spammed with emails offering membership in editorial boards and soliciting submissions.

In 2009, Phil Davis reported that he and a colleague had submitted a grammatically correct but nonsensical manuscript generated by a software program to Bentham’s *Open Information Science Journal* and that he had subsequently received a mail stating that the article had been accepted for publishing provided he would first pay the publication charge of US $800. After some media coverage of the scandal, the editor-in-chief of the journal resigned, claiming that he had no knowledge of the manuscript in question and its acceptance [40].

An example of Dove Press journals is the *International Journal of General Medicine*. Articles in this journal have a very professional visual look. Highly visible statistics on the journal home page promise that the average time from submission to acceptance (including peer review) is 13 days with an additional wait of 15 days until final publication (as of the February 12, 2011).

Bentham and Dove Press seem to have identified a niche market of academic authors who are in desperate need of rapidly getting manuscripts of possibly questionable scientific quality published in journals, which can be labeled peer-reviewed and who are willing to pay the required article processing charges. It is still not clear if either of these publishers will succeed in making this a profitable and sustainable operation.

### Converting Journals From the Subscription Model to Open Access

The vast majority of OA journals are either newly created electronic-only journals or established journals, which make their electronic versions available but finance their operations with income from their printed versions. Converting a subscription journal to full OA is much riskier, particularly if the journal will be funded with APCs, and for this reason, there are few such cases. One example of a successful transition is the Oxford University Press (OUP) journal *Nucleic Acids Research*. The conversion in 2004 was part of broader OA strategy in which a number of OUP journals started allowing authors to open up individual articles in subscription journals against payment [41]. *Nucleic Acids Research* was chosen for the conversion because it was already well established as a quality journal (currently in the top 10% of its field with an ISI impact factor of 7.4); hence, the risk of submissions dwindling away after the conversion to APC funding was deemed to be low.

A totally different type of conversion might come about through pressure from major subscribers. A number of the biggest nuclear research institutes in the world, including the European Organization for Nuclear Research (CERN), have founded the Sponsoring Consortium for Open Access Publishing in Particle Physics (SCOAP3), which aims to force the major physics journals to switch to the open access model. The consortium is currently collecting pledges from potential additional consortium members and by December 20011 had collected 80% of the €10 million they estimate will be required to buy the OA publishing services of the journals in question. In particle physics, a few huge laboratories contribute a major part of the subscription income of the leading journals in the field. The participants in the consortium would offset their contributions by canceling their subscriptions to the targeted journals.

### Experimenting With Gradual Introduction of OA by Introducing Hybrid Journals

Due to the high commercial risks in converting established journals to OA, several major publishers have introduced so-called hybrid journals, traditional subscription journals that allow authors—for a payment—to make their individual articles openly accessible. From the publisher’s viewpoint, this has been a risk-free experiment with OA. Springer pioneered this in 2004 with an open access program known as Open Choice, and other publishers that have followed include Oxford University Press with an open access program know as Oxford Open and Sage with an open access program known as Sage Open. Springer’s initial choice of US $3000 as a uniform price for Open Choice across all journals seems to have set a price standard followed by others. The low uptake of the hybrid model, 1% to 2 % of eligible articles [24], indicates both that the level of the charges might have been too high compared to the benefits the authors perceive they get and also that a uniform pricing model across a large portfolio of journals doesn’t work.

A recent development in the last couple of years is that major mainstream publishers have also started launching new full OA journals. In addition to the purchase of BioMed Central, Springer has, for instance, recently launched 32 full open access journals under the label Springer Open.

### Mega Journals

A new type of journal that has emerged recently is the *Mega* journal, publishing several thousand articles per year over a broad spectrum of topics. The primary example of this type is *PLoS ONE*, which accepts manuscripts in any field of science or medicine. In addition to the broad scope, *PLoS O* *NE* introduced an important change to the function of the peer review. This change is best explained by a direct quote from the journal web site [42]:

Too often a journal's decision to publish a paper is dominated by what the editor/s think is interesting and will gain greater readership—both of which are subjective judgments and lead to decisions which are frustrating and delay the publication of your work. PLoS ONE will rigorously peer review your submissions and publish all papers that are judged to be technically sound. Judgments about the importance of any particular paper are then made after publication by the readership (who are the most qualified to determine what is of interest to them)

To support the idea that it is the readers that eventually will determine the importance and the *contribution* of any particular article, *PLoS O* *NE* utilizes interactive tools for readers and metrics such as downloads and citations per paper. The download statics show a highly skewed distribution between a vast majority of articles that have less than average readership and a small minority of articles that are widely read and cited. *PLoS O* *NE* thus seems to be succeeding in combining the dissemination function of a subject-based preprint repository such as arXiv and the quality certification function of traditional journals. Since 2010, *PLoS O* *NE* has had an impact factor of 4.3. In only five years, it has rapidly increased its publication volume to over 10,000 articles per annum.

Recently, several mainstream science publishers have launched this type of journal, for instance, *Sage Open* for the social sciences and humanities, *Nature Scientific Reports* for the natural sciences, *BMJ Open* for medicine and the Royal Society´s *Open Biology*.

*Acta Crystallography: Structure Reports Online* represents a totally different type of mega journal. Published by the International Union of Crystallography, it was originally, like a number of other journals, a subscription journal but converted to OA in 2008 [43]. The APC is low at only US $150 as compared with US $1300 at *PLoS O* *NE*. *Acta Crystallography: Structure Reports Online* publishes short, highly structured articles in an extremely narrowly field. As the publication volume has increased (over 5000 articles in 2008) the journal archive has begun to look more and more like a database of scientific data.

*Acta Crystallography: Structure Reports Online* also highlights one aspect of publishing where the difference between subscription and OA journals is accentuated. In subscription journals, the rights of authors and readers are highly restricted in order to protect the commercial interests of the publishers. In OA journals, there is no such interest at stake, and publishers mostly allow the authors to retain the copyright. Furthermore, OA publishers have increasingly started to adopt creative commons (CC) licenses, which quite explicitly regulate the rights of readers (including software tools) to use and reuse the publications and which include computer readable versions as a standard feature. The CC licenses are related to the licenses (ie, GNU) used for open-source software.

The CC licenses are vital in promoting the reuse of published research data, in particular in the sciences. *Acta Crystallography: Structure Reports Online* *,* for instance, allows authors to include structured data sets in the refereed articles, using a standardized syntax called crystallographic information file (CIF). Murray-Rust [44] has demonstrated how such data from uncoordinated articles found on the Web can subsequently be harvested by data mining tools to form a knowledge base of much greater power than the isolated articles.

Other publishers are also experimenting with linking journal articles and datasets. *Human Genomics and Proteomics* *,* which started publishing in 2009, is a joint venture of Sage and Hindawi and encourages authors to publish data sets that will be stored in an open repository called FINDbase, a population-specific genetic database that charts causative mutation frequencies and their associated disorders in several countries around the world [45]. An author can submit the dataset and an abstract about it for peer review in *Human Genomics and Proteomics* [46]. After acceptance, the abstract is published in the journal. The Genetics Society of America has also recently announced a new OA journal called *G3: Genes|Genomes|Genetics,* which particularly aims to encourage the inclusion of large structured data sets in the articles.

## Discussion

### Different Types of Innovative Features

The journals discussed in this article provide evidence of the opportunities for innovation that open access provides. The rest of this discussion focuses on the different business models used to achieve sustainability for OA publishing and on additional features made possible or facilitated by OA and the electronic format. Table 3 below shows the innovative features discussed earlier in this article in the Introduction as well as which journals are particularly good examples of the use of the feature in question. This does not mean that the journals listed don’t also have the feature in question, for instance, most of the born-OA journals have faster publication schedules than traditional journals. The central innovation has, of course, been the open access as such, and most OA journals have primarily focused on achieving this, with few changes in article formats, peer-review practices, and so on.

**Table 3:** Innovative features discussed in the article and journals that provide good examples of each feature.

Innovative Feature	Example Journals
Universal accessibility	All
Cost-savings by using volunteers	*Elore, Information Research*
Cost savings by using open-source software	*Medical Education Online, Information Research*
Cost savings by using third party e-portals	*Boletim do Museu Paraense Emílio Goeldi*
Funding by article processing charges	Journals from Plos, BMC, Bentham, and DovePress, and the following: *Journal of Medical Internet Research, Medical Education Online, Nucleic Acids Research*
Broader or narrower journal topics due to the global reach	*PLoS ONE, Journal of Negative Results in Biomedicine*
Novel peer-review methods	*Electronic Transactions on Artificial Intelligence, Hydrology and Earth System Sciences, PLoS ONE, Journal of Medical Internet Research*
Faster article publication cycles	*International Journal of General Medicine, PLoS ONE, Journal of Medical Internet Research*
More flexibility in the layout and structure of articles	*The International Journal of Design Computing, Diagnostic Pathology*
Interactivity for after-publication discussions	Journals from PLoS and BMC
Easy reusability of the digital content	*Acta Crystallography: Structure Reports Online, Human Genomics and Proteomics*

### New Ways of Saving Costs and Getting Revenue

Many well-established print journals have been able to rely on their stable subscription income to open up their electronic versions for no charge. This option has been quite common among society journals, in particular among journals from non-English-speaking countries, which in many cases have been able to use national e-portals for free.

There are a number of ways of financing electronic-only OA journals. Many of the early pioneering journals relied on voluntary labor and use of the website of the editor’s university free of charge. This has worked quite well for journals handling small numbers of submissions and publishing on technically simple websites but has not worked well as the number of articles has increased. There are examples of successful early journals that have later adopted article-processing charges in order to ensure continuity and a more professional way of operating. Many independent and society journals have adopted Open Journal Systems software as a cost-effective way of getting a fairly robust IT platform that also incorporates manuscript-handling features.

As professional OA publishers have entered the field, the article processing charge has become the central funding mechanism for large-scale full OA publishing. More and more, it is the quality level of the journal that determines the article processing charge that authors (and their funders) are willing to pay, especially when considering alternatives inside one’s research discipline. In addition to quality, the subject field also affects the possibilities and willingness of authors to pay. APCs in biomedicine are typically higher than in the social sciences, for example.

OA journals that have adopted article-processing charges have almost exclusively levied these for published articles. This means that authors whose manuscripts have been accepted have indirectly paid the costs incurred for rejected manuscripts. This choice has obviously been based on the assumption that charging even a small amount for submissions might stem the inflow of manuscripts.

Normally APCs are the same for all articles within a given journal, but a nice feature of the major OA publishers is that they usually promise to waive the charges for authors who can document that they have problems financing APCs, especially authors from developing countries.

### Broader or Narrower Journal Topics Due to Global Reach

Open access seems to be particularly well suited to what could be called “microtopics” and “macrotopics.” Open access has clearly lowered the threshold for founding new journals in narrow areas, which in the print and subscription model would not have been economically viable. Understandably, many journals founded in the 1990s specialized in topics related to IT and the Internet.

Likewise, open access offers an excellent way for journals from countries outside the major Anglo-Saxon sphere, both those publishing in English and those publishing in other national languages, to increase their readership and impact. Hence, OA lowers the digital divide by allowing scientists in developing nations both better access to mainstream science and increased chances of being read outside their own countries.

A recent trend is the emergence of mega journals, the topics of which span substantial parts of all of science. Of the journals considered, mega journals were published by well-established, credible publishers with professional staff and ready IT infrastructure. The key issue for such journals in particular is the ability to attract submissions, manage reviews, and recruit reviewers.

### Novel Peer-Review Methods

Several OA journals have experimented with different variations of open peer review, which relies on the activity of readers to actively upload comments to the journal websites and which allows the research results to be made public at the preprint manuscript stage. So far, the results are inconclusive, and open peer review is still quite rare.

There is an obvious temptation for some commercial OA publishers so set up journal collections that can publish submissions with minimal costs and efforts for the peer review. While this is fully legal as a business model, the scientific community can ignore giving much credit to such publications in its evaluations. Early evidence also suggests that such publishers have had problems getting enough submissions.

The *review of scientific rigor only*, a concept that *PLoS ONE* has pioneered, and where the scientific contribution is determined by readership and citations rather than the judgment of a couple of peer reviewers, seems on the other hand a very useful innovation, at least as a model for mega journals with broad scope.

### Faster Article Publication Cycles

Faster publication has always been an advantage of open access journals, in particular for journals that are not published in an issue format. Some journals have recently streamlined their processes in order to achieve very short average lead times from submission to publication (of accepted papers). The very short average processing times announced by Dove Press, however, raise many questions concerning the quality of the review process.

The electronic-only format freed OA journals from the straightjacket of the journal issue, and many OA journals have from the start opted for publishing articles on the fly as the articles become technically ready. This was seen early on as one of the major benefits of OA since it speeded up publication, usually by several months. Lately, traditional journals have partly followed this lead by making *articles-in-press* available to subscribers on their websites.

### More Flexibility in the Layout and Structure of Articles

The electronic format has also opened up new possibilities for including types of presentation formats other than the linear text format, particularly in OA journals, which don’t have the burden of also being published in print. Media enhancements as well as documentation attached to the articles have also been tried, but such additions may present problems for peer reviewing and can be tricky to handle for journal archives. On the whole, authors seem, however, to be rather conservative concerning changes from the traditional look and feel of articles.

Of the different types of material that could not easily fit into traditional printed articles, the structured data set is the most promising, particularly in some domains of science where such data can be data mined and harvested into bigger aggregate services.

### Interactivity for After-Publication Discussions

The electronic format offers opportunities for new kinds of interactive functionality, which was not possible in printed journals. Since OA journals were the first electronic scholarly journals, it was natural that they first started to experiment with reader comments, open peer review, and so on. Other interesting features that are included nowadays in PLoS journals, for instance, are download and citation metrics. Subscription journals can also include such features now, but only in the electronic versions.

### Easy Reusability of the Digital Content

For open access journals, the assignment of copyright and the licensing agreements for readers and automated tools differ radically from traditional subscription-based journals. During the 1990s, the OA journals were mostly just *open* and the copyright and license terms were usually not formalized. The Creative Commons standard licenses for Web material emerged after the year 2000 and are eminently suited for scientific publications and data. Currently, most professional OA publishers use some form of CC license, and its use is also spreading among independent OA journals. The increasing use of Creative Commons licenses in OA journals facilitates, in particular, the data mining of data attached to articles.

### Conclusions

Open access publishing is rapidly increasing its share of the overall volume of scientific journal publishing with an annual growth rate of 20% and an estimated number of more than 250,000 articles in 2011 (extrapolated from [10]). So far, this growth has almost exclusively come from independent, society, and newly started OA publishers. Now the tide seems to be turning. The fact that major scholarly publishing companies are in the process of launching new APC funded journals is a clear indication that they have judged that the OA model has proved to be sustainable. Existing OA journals have already tried out many new ideas in scholarly publishing, as reported in this paper. The successful innovations are fast becoming part of the academic infrastructure, with scientists voting with their manuscripts as to which ones will prevail.

## Abbreviations

APC: article processing charge
BMC: BioMed Central
CERN: European Organization for Nuclear Research
SCOAP3: Sponsoring Consortium for Open Access Publishing in Particle Physics
CC: creative commons
CIF: crystallographic information file
HTML: hypertext markup language
ISI: Institute for Scientific Information
IT: information technology
J-STAGE: Japan Science and Technology Aggregator, Electronic
OA: open access
OJS: Open Journal Systems
OUP: Oxford University Press
Redalyc: Red de Revistas Cientificas de América Latina y el Caribe
SciELO: Scientific Electronic Library Online
SSHRC: Social Sciences and Humanities Research Council
